# Comparison of chromosomal and array-based comparative genomic hybridization for the detection of genomic imbalances in primary prostate carcinomas

**DOI:** 10.1186/1476-4598-5-33

**Published:** 2006-09-04

**Authors:** Franclim R Ribeiro, Rui Henrique, Merete Hektoen, Marianne Berg, Carmen Jerónimo, Manuel R Teixeira, Ragnhild A Lothe

**Affiliations:** 1 Department of Genetics, Portuguese Oncology Institute – Porto, Porto, Portugal; 2 Department of Cancer Prevention, Institute for Cancer Research, The Norwegian Radium Hospital, Oslo, Norway; 3 Department of Pathology, Portuguese Oncology Institute – Porto, Porto, Portugal; 4 Department of Pathology and Molecular Immunology, Institute of Biomedical Sciences, University of Porto, Porto, Portugal; 5 Fernando Pessoa University, Porto, Portugal; 6 Department of Molecular Biosciences, University of Oslo, Oslo, Norway

## Abstract

**Background:**

In order to gain new insights into the molecular mechanisms involved in prostate cancer, we performed array-based comparative genomic hybridization (aCGH) on a series of 46 primary prostate carcinomas using a 1 Mbp whole-genome coverage platform. As chromosomal comparative genomic hybridization (cCGH) data was available for these samples, we compared the sensitivity and overall concordance of the two methodologies, and used the combined information to infer the best of three different aCGH scoring approaches.

**Results:**

Our data demonstrate that the reliability of aCGH in the analysis of primary prostate carcinomas depends to some extent on the scoring approach used, with the breakpoint estimation method being the most sensitive and reliable. The pattern of copy number changes detected by aCGH was concordant with that of cCGH, but the higher resolution technique detected 2.7 times more aberrations and 15.2% more carcinomas with genomic imbalances. We additionally show that several aberrations were consistently overlooked using cCGH, such as small deletions at 5q, 6q, 12p, and 17p. The latter were validated by fluorescence in situ hybridization targeting *TP53*, although only one carcinoma harbored a point mutation in this gene. Strikingly, homozygous deletions at 10q23.31, encompassing the *PTEN *locus, were seen in 58% of the cases with 10q loss.

**Conclusion:**

We conclude that aCGH can significantly improve the detection of genomic aberrations in cancer cells as compared to previously established whole-genome methodologies, although contamination with normal cells may influence the sensitivity and specificity of some scoring approaches. Our work delineated recurrent copy number changes and revealed novel amplified loci and frequent homozygous deletions in primary prostate carcinomas, which may guide future work aimed at identifying the relevant target genes. In particular, biallelic loss seems to be a frequent mechanism of inactivation of the *PTEN *gene in prostate carcinogenesis.

## Background

Prostate cancer is a frequent and heterogeneous malignancy with few established prognostic markers. Increased knowledge on the genetic basis of this condition is expected to significantly improve the clinical management of these patients. Most of the genetic data currently available on this malignancy has been obtained using chromosomal comparative genomic hybridization (cCGH), a whole-genome screening methodology well established in the scientific field [1]. We have recently published a statistical dissection of the cCGH data available in the literature and proposed two main genetic pathways involved in prostate carcinogenesis, starting either with 8p or 13q deletions [2]. We showed that 8q gain and 13q loss were good predictors of progression into locally invasive disease and that losses of 6q and 10q were significantly associated with metastatic cancers. In addition, some of these genetic changes have shown prognostic value independently of tumor grade and stage [3-6].

The recent advent of microarray-based platforms for the detection of genome-wide copy number changes promises to uncover novel recurrent genetic aberrations and provide a more accurate delineation of genomic regions previously known to be altered in different cancer types. However, there is still no consensus regarding the scoring of array-based comparative genomic hybridization (aCGH) results, making it difficult to objectively compare findings obtained by different platforms and analysis tools. A few aCGH studies of prostate cancer cell lines have been reported [7-11], but most cell lines grow as stable, uncontaminated cell populations with clonal karyotypes. This makes the comparison of different platforms and scoring methods easier than for clinical samples, which often contain varying degrees of non-neoplastic cell contamination and thus fail to show the fluorochrome ratio intensities expected for low-level copy number changes. Whole-genome aCGH findings have been reported in small subsets of primary prostate carcinomas [12-14], and high-resolution platforms have been developed to study recurrently affected genomic regions [14,15]. However, Paris *et al*. were the first to use the aCGH methodology to study a larger series of clinical prostate cancer samples [16,17]. The particular scoring methodology used in those studies resulted in the detection of a large percentage of single clone alterations of unclear significance. Furthermore, the concordance between the previously established chromosomal CGH and the new array-based CGH platforms could not be conclusively evaluated, since genetic information obtained with the former method was available only for a small subset of the samples.

In the present study, we systematically compared aCGH and cCGH profiles of 46 primary prostate carcinomas and determined the best aCGH scoring methodology to delineate genomic copy number changes relevant for prostate carcinogenesis.

## Results

### Quality control

Clones that failed to produce a result in more than 60% of the sample set were removed from further analysis, as were those displaying copy number changes in at least two negative controls. Clones with known polymorphic regions were not present in the array. Additionally, analysis of the dye-swap experiments and negative controls suggested a dye-specific affinity of several clones on chromosome X and Y, which are rich in repetitive sequences. As these seemed to produce copy number aberrations (not previously detected by cCGH) in all samples, we chose to remove them from the analysis. From the 3568 clones in the microarray, 2787 passed these stringent quality criteria. The median percentage of clones remaining per sample (out of 2787) was 97% in the negative controls, 96% in the biopsy samples and 89% in the prostatectomy series.

### Comparison of scoring methods

**Figure 1:**
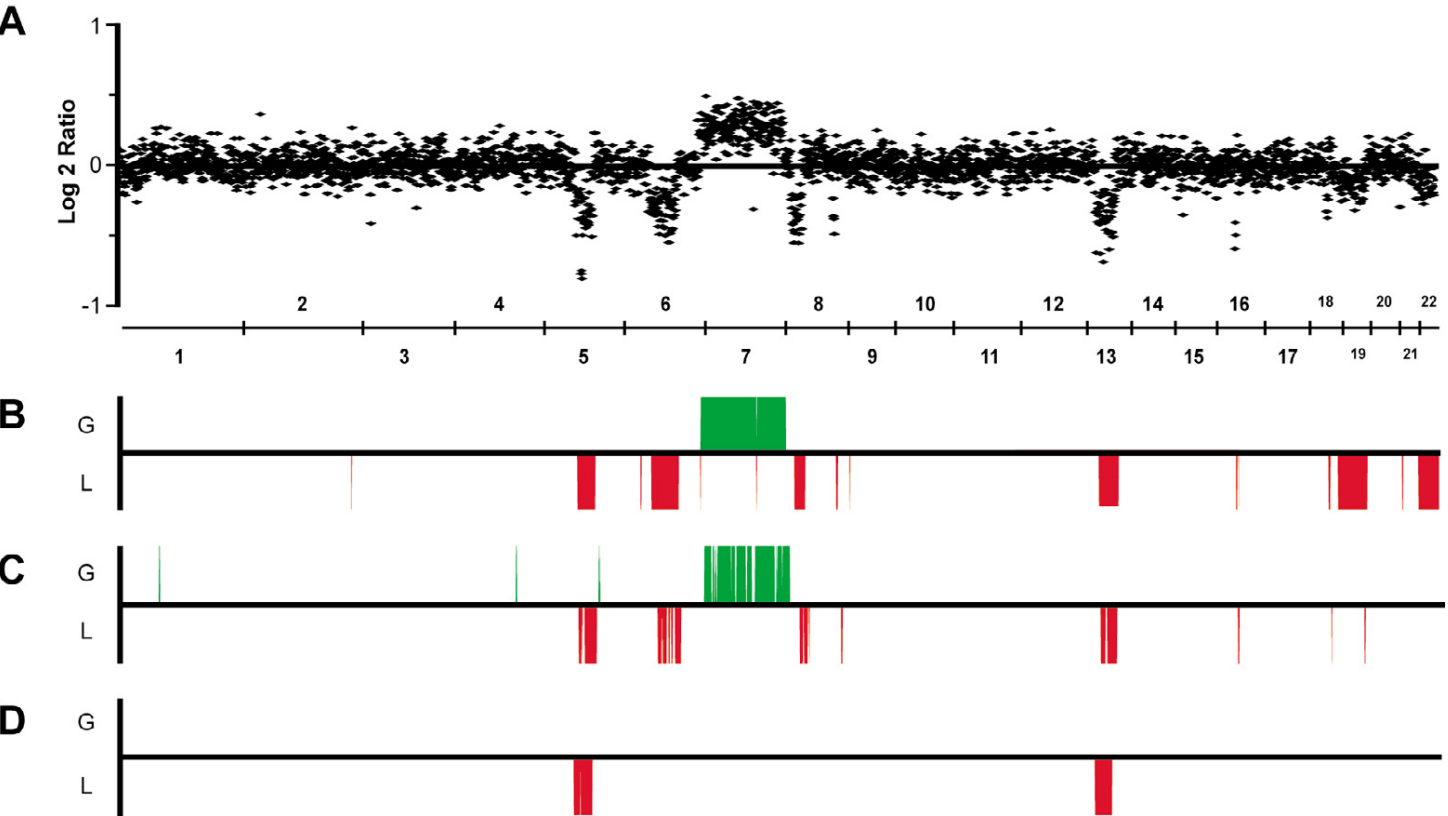
**Comparison of aCGH score results for sample "Bp22" using different automated scoring approaches**. (**A**) Normalized log-2 ratios, with clones ordered according to their genomic position. Note that the theoretical intensity values for gains and losses are not reached. (**B**) Results using aCGH-Smooth. (**C**) Results using sample-specific fixed thresholds calculated in Normalization Suite. (**D**) Results using CGH-Plotter. For the purposes of visualization and comparison, all diagrams were generated in Microsoft Excel based on data provided by the different analysis tools, and thus do not correspond to the visual outputs provided by each individual software.

Sample-specific fixed-thresholds, even when stringently determined, provided a fragmented genetic profile in which several low-level copy number changes (CNCs) were not scored and a large number of single clone aberrations (average of 5.8 per sample) as well as false positive findings (average of 3.7 CNCs per control) were obtained. The data segmentation approach provided by CGH-Plotter was also affected by low intensity ratios, resulting in most gains and several deletions, confirmed to be present using cCGH, being missed. On the other hand, the number of single clone aberrations (0.5 per sample), as well as false positive findings (0.1 CNCs per control), was greatly reduced. aCGH-Smooth, by focusing on the detection of contiguous groups of clones with similar mean intensities, was able to score a large number of gains and losses with intensities that did not reach theoretical ratios for a stroma-free tumor sample. This strategy thus detected twice as much CNCs than the previous ones, with the advantage of producing very few single-clone aberrations (average of 1.4 per sample) and virtually no false positive findings (average of 0.1 CNCs per control). Due to their uncertain significance, the few single clone aberrations were not included in the final scoring. Figure 1 provides a schematic representation of the individual profiles produced by the three aCGH scoring methodologies tested on a sample with known copy number aberrations.

### Comparison between cCGH and aCGH findings

**Figure 2:**
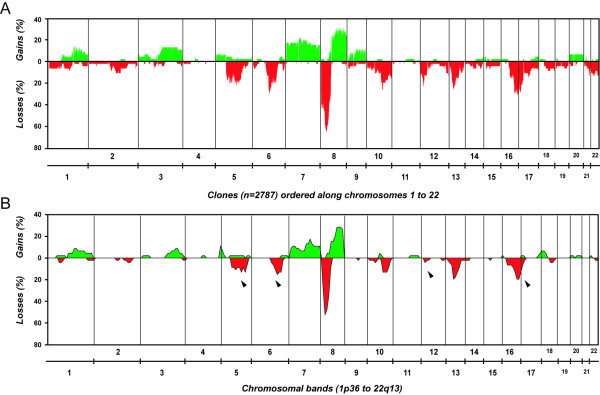
**Genomic findings in 46 primary prostate carcinomas**. (**A**) Array-CGH findings (38 samples with copy number changes). (**B**) Chromosomal CGH findings (31 samples with copy number changes). Arrows indicate relevant differences in the frequency of aberrations detected by both techniques.

aCGH confirmed 95% of the 146 copy number changes detected by cCGH in the 46 prostate carcinomas (Figure 2). Most of the non-confirmed aberrations involved single chromosomal bands located at chromosomal ends. Seven cases without copy number changes by cCGH were found to have genomic aberrations upon aCGH analysis, representing a 15.2% detection increase of abnormal cases. Regarding individual aberrations, aCGH detected 2.7 times more copy number changes than cCGH (347 versus 146). Forty-five percent of the gained regions spanned more than 50 clones, whereas a large proportion of lost regions involved 20 to 50 clones (33.8%). Overall, 73.2% of all gains and 70% of all losses involved at least 10 clones, which corresponds roughly to the 10 Mb resolution level estimated for cCGH. Up to 60% of the gains and 50% of the losses larger than 10 clones had been detected using cCGH. Specifically, deletions of 5q (*p *= 0.066), 6q (*p *= 0.065), 12p (*p *= 0.014), and 17p (*p *= 0.072) were particularly overlooked by cCGH, whereas deletions at 8p and gains of 7 and 8q were detected by both techniques in almost identical proportions.

### aCGH profile of prostate carcinomas

**Figure 3:**
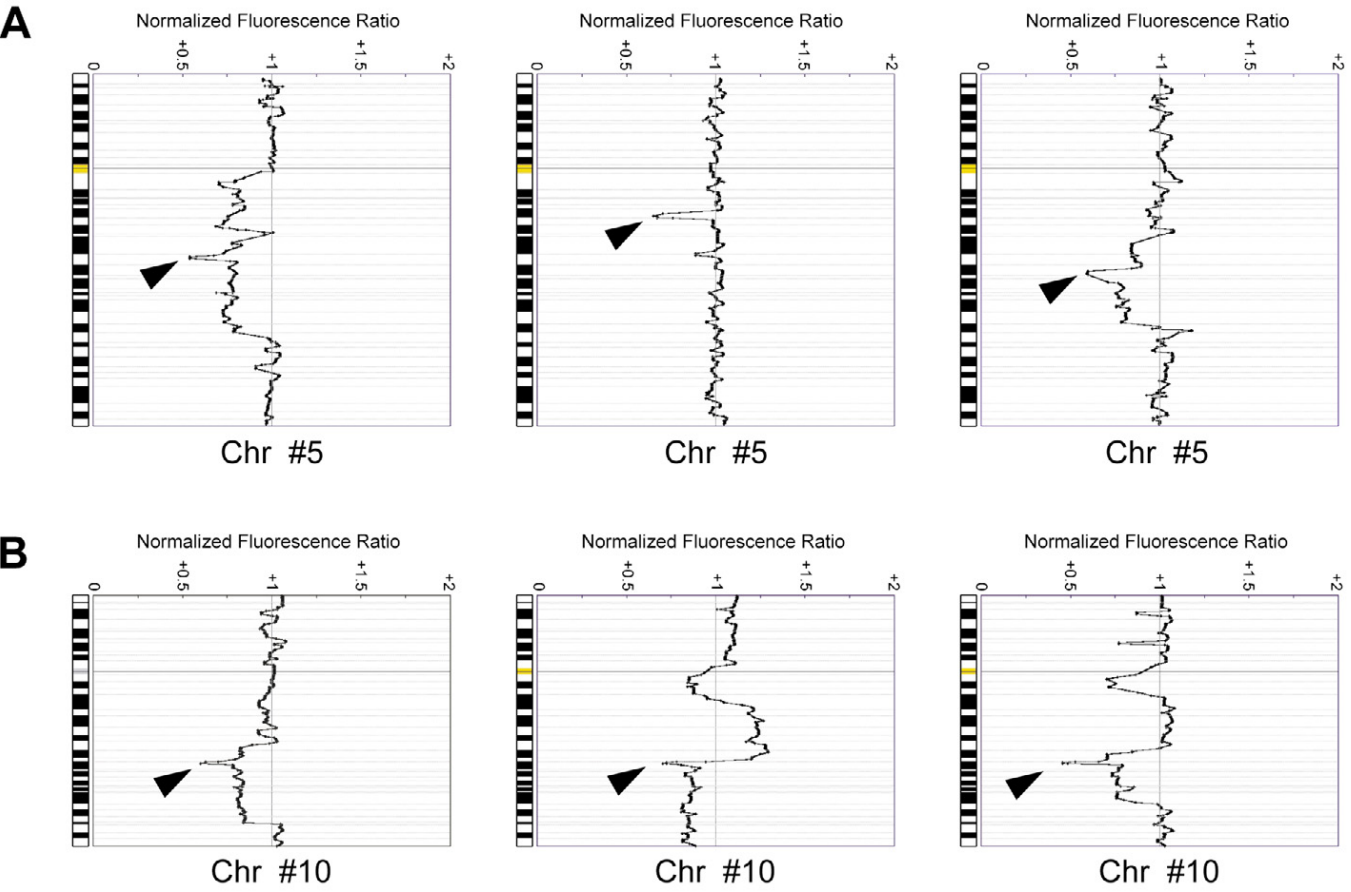
**Examples of homozygous deletions revealed by aCGH (arrow heads)**. (**A**) Homozygous deletions at different regions of 5q. (**B**) Recurrent homozygous deletions at 10q23.31, encompassing the *PTEN *gene region.

Overall, 38 cases (83%) displayed copy number changes, with losses of genomic material being 2.7 times more frequent than gains (Figure 2, Table 1). Seven out of 15 cases without copy number changes by cCGH were found to harbor aberrations upon aCGH analysis. Interestingly, 7/24 prostatectomy and 1/22 biopsy samples did not display copy number changes even at this level of resolution. Regions of recurrent genomic loss were located at 8p (67%), 5q (39%), 16q (37%), 6q (35%), 13q (33%), 10q (33%), 17p (30%), 12p (24%), and 2q (20%), whereas frequent copy number gains were observed at 8q (30%), 7 (22%), and 3q (13%) [see Additional file 1]. Amplifications were detected in a total of four biopsy samples (Table 2), whereas homozygous deletions were detected in a total of 10 carcinomas (Figure 3, Table 2), most frequently at 10q23.31 (seven out of 12 tumors with 10q loss showed homozygous deletion at this locus, involving a minimum common region of 4 clones).

**Table 1:** Overview of aCGH findings in 46 prostate cancer samples

	Frequency (n = 46)	SRO^1^	Size (Mb)	Genomic position (Mb)
Losses				
8p	67.4%	8p21.2-8p22	12.0	15.36–27.36
5q	39.1%	5q13.1 5q13.2-5q13.3 5q21.1 5q22.2-5q23.1	0.6 2.1 2.0 7.7	66.79–67.41 71.74–73.87 99.03–101.09 112.07–119.75
16q	37.0%	16q12.1-16q12.2 16q22.2-16q22.3 16q23.3-16q24.1	4.1 1.7 3.6	49.70–53.86 70.70–72.37 81.20–84.83
6q	34.8%	6q14.1-6q14.3 6q16.2-6q22.31	3.5 23.6	82.59–86.12 99.38–123.02
10q	32.6%	10q23.31 10q26.3	1.0 2.6	89.69–90.69 130.01–132.63
13q	32.6%	13q14.1-13q14.3 13q21.32-13q21.33	5.5 4.7	39.49–45.03 66.42–71.16
17p	30.4%	17p13.1-17p13.2	2.3	6.14–8.49
12p	23.9%	12p13.2	1.9	10.93–12.86
Gains				
8q	30.4%	8q11.21-8q12.1 8q13.2-8q24.23	10.8 68.2	48.76–59.73 68.36–136.56
7q	21.7%	7q11.21-7q11.22 7q21.11-7q22.1	6.3 19.5	64.63–71.01 80.26–99.76

Aberrations occurring in less than 20% of the samples are not displayed.

^1 ^Smallest region of overlap (often more than one per chromosomal arm).

**Table 2:** Amplifications and homozygous deletions detected in 46 prostate cancer samples

	**Cytoband (n° cases)**	**Size (Mb)**	**Genomic position (Mb)**
Homozygous deletions			
	5q13.1 (1)	0.62	66.79–67.41
	5q15 (1)	0.97	93.62–94.59
	5q21.1-5q21.2 (1)	2.01	101.08–103.09
	10q23.31 (7)	1.10	89.60–90.70
	11q23.2-11q23.3 (1)	1.10	113.80–114.90
Amplifications			
	6q24.1-6q25.3 (1)	13.80	142.29–156.09
	7q11.22-7q11.23 (1)	5.90	68.97–74.87
	7q22.1 (1)	0.72	98.59–99.31
	8p12 (1)	1.96	36.43–38.39
	8q22.2-8q22.3 (1)	3.17	101.35–104.52
	8q23.2-8q24.22 (1)	23.83	111.82–135.65
	11q22.3-11q23.1 (1)	3.81	107.37–111.18
	17p11.2 (1)	0.99	19.18–20.17
	17q23.2-17q23.3 (1)	1.40	56.75–58.15
	19p13.3 (1)	0.98	5.63–6.61

### FISH and mutation analyses of TP53

**Figure 4:**
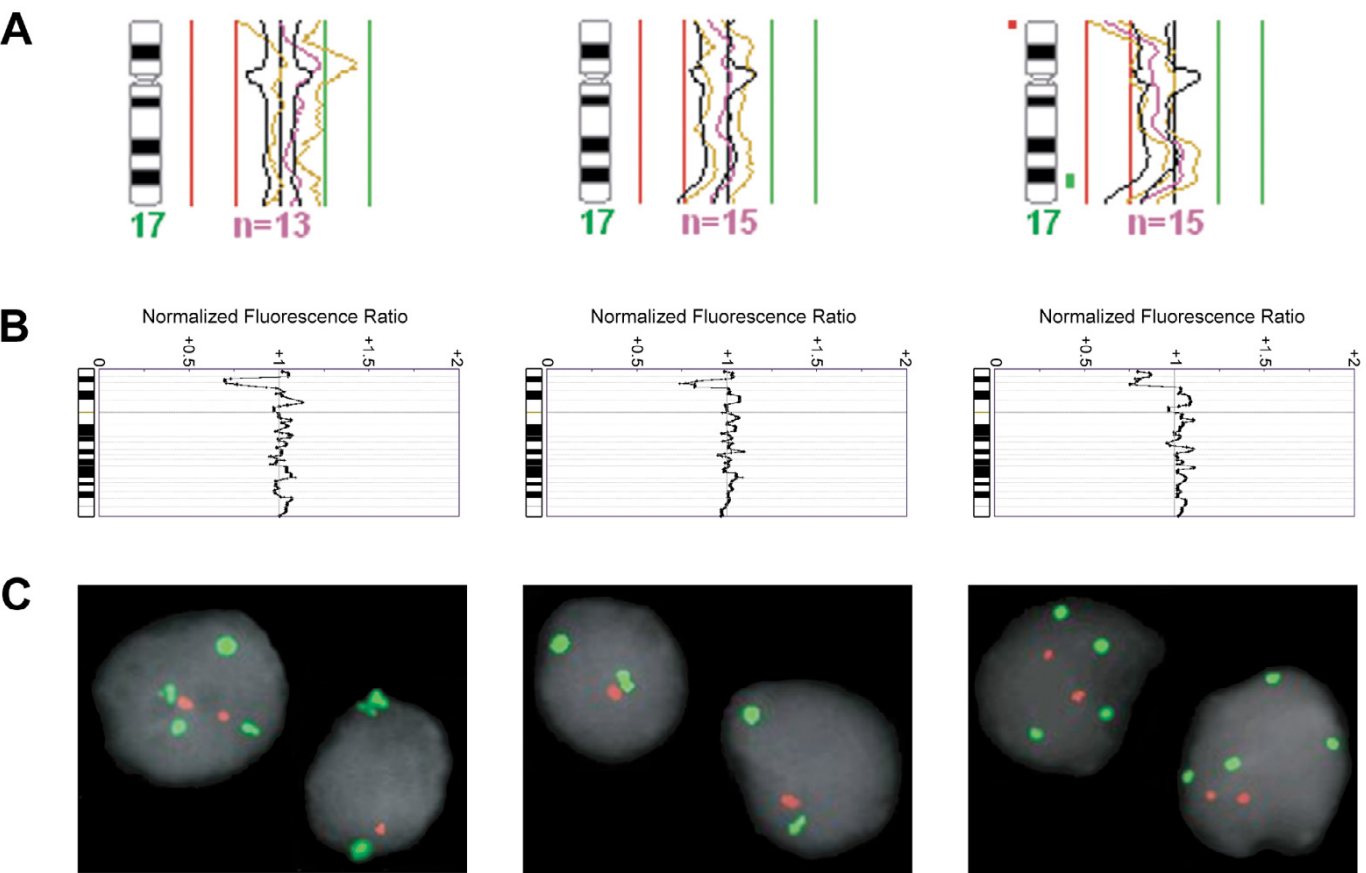
**Genomic findings using cCGH, aCGH, and FISH in three selected biopsy samples**. (**A**) cCGH results for chromosome 17, with a terminal 17p13 deletion detected in only one case. (**B**) aCGH findings showing loss of 17p13 in all three cases. (**C**) FISH findings confirming the loss of one or more *TP53 *copies (red) compared to the centromeric probe for chromosome 17 (green) in all three cases.

Hybridization was successful in all 10 paraffin embedded core biopsies analyzed by dual-color FISH. These corresponded to samples with (n = 7) and without (n = 3) deletions at 17p13 detected by aCGH. FISH results confirmed the loss of one or more copies of the *TP53 *probe (compared to the control probe) in all but one of the cases with 17p loss (Figure 4; the exception was a case deemed uninformative due to the small size of the paraffin section analyzed). The remaining three samples displayed a normal fluorescent pattern with two signals for both the centromeric and the 17p probes. Regarding *TP53 *mutation screening of the 51 samples, including the nine with 17p losses by cCGH, aCGH, or FISH, only one mutation (exon 5, codon 177, CCC->CTC, Pro-> Leu) and a known polymorphism (exon 6, codon 213, CGA->CGG, Arg->Arg, detected in four samples) were present, all in cases without a 17p13 deletion.

## Discussion

In this work we used array-based CGH to assess the genomic profile of a large series of primary prostate carcinomas. As these samples had previously been analyzed using chromosomal CGH, we were able to compare the two techniques in terms of sensitivity and overall performance, and to test distinct automated scoring approaches for aCGH data. Whereas most scoring methods will achieve concordant results if a given sample is pure and the hybridization quality is excellent, clinical samples usually contain non-neoplastic cell populations that influence the interpretation of the results. In the particular case of the prostate gland, the enriched cellular content of the stromal component should not be underestimated. Combined with the variability within chromosome spreads (in cCGH), labeling efficiency, and hybridization behavior, a certain level of methodological noise/variability is expected that may seriously influence the analysis. In a recent paper by Lai *et al*. [18], several automated scoring methodologies were compared using datasets recreating distinct aberrations and background noise, and only a few were able to reliably score low-level copy number changes. Taking this information into account, and using our cCGH data as a starting point, we compared three freely available analysis tools representing common aCGH scoring methodologies. We found fixed-thresholds to be extremely affected by the quality of the hybridization and the presence of normal cells, which resulted in known alterations being missed completely or scored only partially. The large number of single-clone aberrations obtained also rendered the distinction between true copy number changes and false positive results subject to interpretation and additional validation. The data segmentation approach of CGH-Plotter produced minimal levels of single clone aberrations, but was unable to detect most low-intensity changes. Finally, aCGH-Smooth consistently detected low-level copy number changes with only residual levels of single-clone aberrations and false positive findings, thus providing a more sensitive and reliable approach to the scoring of our 1 Mb BAC array data.

Using this analytical tool, 95% of the changes detected using cCGH were confirmed by aCGH. The theoretical 10-fold increase in resolution of aCGH resulted in an increase of 15.2% in the proportion of genetically abnormal prostate carcinomas and in the detection of 2.7 times more copy number aberrations. Strikingly, of the aberrations involving more than 10 Mb (the estimated resolution limit of cCGH), 45% had not been scored using cCGH. We believe this discrepancy reflects two limitations of cCGH, namely the lack of sensitivity in detecting low-intensity alterations (independently of the size of the aberration) and the inherent difficulty in scoring regions of metaphase chromosomes of smaller size and variable hybridization behavior (17p, 18p, 19, 20, 21, and 22). It is noteworthy that deletions at 8p and gains at 8q and 7 were equally detected by both methodologies, whereas deletions at 5q, 6q, 12p, and 17p were particularly overlooked using cCGH. Paris et al. [17] have previously reported such a comparison in a series of 20 formalin-fixed paraffin embedded prostate cancers. In their work, 90% of the cCGH copy number changes were confirmed by aCGH, which detected ~3.4 times more alterations. As they used fixed thresholds to score their data, however, 44% of the aCGH findings consisted of single clone aberrations, thus requiring careful interpretation and validation.

The overall profile obtained for our prostate cancer samples was comparable to that described in previous aCGH studies of clinical samples [13,16,17]. It is noteworthy that most gains were detected in the overall more advanced group of carcinomas sampled by biopsy, whereas 77% of the alterations in the prostatectomy series corresponded to deletions, which according to the literature are the most common events in prostate carcinogenesis [2]. Interestingly, seven prostate cancers sampled by prostatectomy (early staged tumors) did not display copy number changes even at this level of resolution, whereas losses at 8p and 16q and gain at 8q were already present in a considerable percentage of clinically confined carcinomas, indicating these alterations arose early during tumor progression. We and others have in fact shown that 8q gain is significantly associated with increased tumor grade and worse patient outcome [3,5,6,19], therefore suggesting that some early cancer foci already carry genetic features of bad prognosis, whereas others do not display copy number changes at all and possibly correspond to a subset of less aggressive or even latent lesions.

Loss of 17p, another alteration previously associated with poor prognosis and frequently overlooked by cCGH, often occurred together with 8q gain in our sample set. This led us to perform FISH and mutation screening for the most likely candidate at this location, the *TP53 *gene. Mutation frequencies for *TP53 *are extremely variable in prostate cancer studies (ranging from 3–45%), but overall it is consensual that most clinically confined tumors have no mutations, whereas metastatic and androgen independent cancers harbor a high frequency of *TP53 *mutations [20,21]. We detected only one mutation in our set of clinically confined carcinomas, which is in accordance with the literature and suggests that this genetic event is more important for the progression, rather than to the establishment, of prostatic carcinomas. Loss of 8p, on the other hand, is an early and frequent finding in prostate cancer with no significant differences between cCGH and aCGH. It involves a minimum region of overlap spanning ~12 Mb (8p21.2 to 8p22) that encompasses over 50 confirmed genes with distinct cellular functions, making it difficult to pinpoint single candidate targets. Our and previous aCGH studies have been unable to find homozygous deletions at this chromosome arm, suggesting that many genes in this region may thus be working together on a dosage dependent manner to induce the initial stages of prostate carcinogenesis [22,23].

Deletions at chromosomal region 10q are also a frequent finding in prostate cancer cells, albeit associated with advanced disease. In 12 out of 15 cases with 10q loss in our series, a common region at 10q23.31 (~1 Mbp long) was affected. Strikingly, in seven of these 12 carcinomas the deletion was homozygous. The only cancer-relevant gene from the few candidates at this location is *PTEN/MMAC1 *[24,25], as it has already been shown that *PTEN *expression is reduced in a large subset of advanced prostate cancers [26,27]. Recent work on mouse models [28-30] suggests that the absence of functional *PTEN *confers proliferating cells the ability to overlook apoptosis even when subjected to apoptotic stimuli. Haploinsufficiency of *PTEN *seems to already have a dramatic influence on the cellular response to apoptosis [28], with the loss of the second allele being actively selected for during disease progression [30]. Interestingly, analyses of this multifunctional protein phosphatase generally describe very low mutations frequencies [31-34], which further indicates that homozygous deletions, rather than mutations or epigenetic silencing, are the major mechanism of gene inactivation at this locus. This hypothesis has been recently strengthened by the recurrent finding of homozygous deletions encompassing the *PTEN *region in several prostate cancer cell lines and xenografts [35,36], as well as in primary tumors [37]. Homozygous deletions affecting 5q were also relatively frequent in our series of primary prostate carcinomas, but these were heterogeneous and the potential target genes remain unknown.

## Conclusion

We conclude that aCGH can significantly improve the detection of genomic aberrations in cancer cells as compared to previously established whole-genome methodologies, although stromal contamination may significantly influence the sensitivity and specificity of most automated scoring approaches. The increased resolution of aCGH revealed several previously undetected aberrations and refined the breakpoints of those already found by cCGH. The recurrent regions of copy number gains and losses in primary prostate carcinomas highlighted in this study, as well as the novel amplified loci and frequent homozygous deletions, may guide future work aimed at identifying the relevant target genes.

## Materials and methods

### Prostate carcinoma samples

We have previously reported the genomic findings detected by cCGH in a series of prostatectomy specimens containing cancer [2] and in a series of fine- needle biopsies from prostate cancer suspects [6]. For the present aCGH study, 24 samples from the former and 22 samples from the latter series were selected, because we wanted to include early-staged tumors, as well as samples from more advanced, genetically complex cancers. From the prostatectomy series, in which all samples contained >70% tumor cells, cases were selected to equally represent different Gleason score categories. From the biopsy series, only samples with morphological evidence of tumor were used and selection was based mostly on DNA availability. From the selected samples, 6/22 biopsies and 9/24 prostatectomies had displayed no copy number changes using cCGH. The same DNA stocks were used for cCGH and aCGH. Additionally, a total of 51 carcinomas for which good quality DNA was available (46 samples from Ribeiro *et al*., 2006a, including the 24 selected for this aCGH study, and 5 biopsy samples from Ribeiro *et al*., 2006b, also included in the present report) were evaluated for *TP53 *gene mutations. Several paraffin-embedded tissue blocks corresponding to biopsy samples analyzed by aCGH were also selected for FISH validation studies.

### Array-based comparative genomic hybridization

#### Clone set

We used the Human 4 k Genome-wide 1 Mb resolution Arrays provided by the Norwegian Microarray Consortium (National technology platform supported by the functional genomics program of the Research Council of Norway [38]). Each slide consists of 3568 BAC/PAC probes positioned along the genome at an average resolution of 1 Mb, printed in duplicate onto two identical blocks in the array, for a total of four replicates per clone. Probe DNA was obtained from the 1 Mb BAC/PAC clone set kindly provided by Dr. Nigel Carter at the Wellcome Trust Sanger Institute, UK [39], amplified using DOP-PCR, and spotted onto CodeLink slides (Amersham Biosciences, Chalfont St Giles, UK) using a MicroGrid II arrayer (BioRobotics, Boston, USA). Mapping information (clone location and cytogenetic bands) was retrieved from the Ensembl Human Genome Browser v36, December 2005 freeze [40].

#### Labeling and hybridization

DNA from the 46 prostate samples had been extracted using standard methods. The same commercially available male control DNA (Promega Corporation, Madison, WI) was used as reference for all samples. For each experiment, 500 ng of test and reference DNA were digested with Dpn II (New England Biolabs, Ipswich, MA), purified using the QIAquick PCR purification kit (Qiagen Inc, Valencia, CA), and labeled with Cy3-dCTP (test) or Cy5-dCTP (reference) (PerkinElmer, Boston, MA) in a random-primer reaction with the BioPrime Array CGH Genomic Labeling Kit (Invitrogen, Paisley, UK). Unincorporated nucleotides were removed using micro-spin G50 columns (Amersham Biosciences, Chalfont St Giles, UK). Labeled DNAs were combined, mixed with 135 μg of human Cot-1 DNA (Invitrogen, Paisley, UK), precipitated using ethanol and ressuspended in hybridization buffer containing 50% formamide, 10% dextran sulphate, 2 × SSC, 4% SDS, and 10 μg/μL yeast tRNA (Invitrogen, Paisley, UK). Samples were denatured at 72°C for 10 minutes and incubated at 37°C for 60 minutes before being hybridized onto the slides in a GeneTAC Hybridization station (Genomic Solutions Ltd, Huntingdon, UK). Hybridization took place over 36 hours, followed by automated post-hybridization washes in 50% formamide/2 × SSC (45°C), 2 × SSC/0.1% SDS (37°C), and PN buffer (37°C). Slides were dried by centrifugation after a brief wash in 0.05 × SSC and scanned with an Agilent G2565BA microarray scanner (Agilent Technologies, Palo Alto, CA). Five control hybridizations (normal male versus normal female DNA) were performed, as well as five dye-swap experiments using randomly selected samples. Data from 11 additional negative controls run during the same period with different batches of reference DNA were kindly provided by the Microarray Core Facility to validate the clone set.

#### Image analysis and processing

Analysis of the microarray images was performed in GenePix Pro 6.0 (Axon Instruments Inc., Foster City, CA), with the median pixel intensities for each channel (with background subtraction) being calculated for each spot. For each sample, Genepix results were exported as a TAB-delimited "GPR" file into Normalisation Suite [41], where background-subtracted channel intensities were normalized (local linear normalization) and combined to produce the final intensity ratios for each feature. For the automated scoring of copy number aberrations, three methods were compared: sample-specific fixed-thresholds, calculated as 2.5 times the baseline noise levels for each sample (Normalisation Suite [42]); a data segmentation approach using K-means clustering (CGH-Plotter [41]), and breakpoint estimation (aCGH-Smooth [43]). The final choice for automated scoring fell upon aCGH-Smooth. Graphical visualization of the log-2 ratios for each sample and the overall results for all samples (clones indexed by their physical location along the genome) were generated in Normalisation Suite and Microsoft Excel, respectively. Amplifications were scored whenever log-2 intensity ratio was larger than 0.75. For determination of homozygous deletions, the average log-2 intensity ratios for deleted regions was calculated for each sample, and clones reaching at least twice this value were scored.

### Fluorescent in situ hybridization

For ten selected biopsy samples, four-micron thick sections from a representative paraffin-embedded block were cut onto SuperFrost Plus Adhesion slides (Menzel-Glaser, Braunschweig, Germany). Sample processing, hybridization, and analysis were performed as described previously [6]. A locus-specific probe for the *TP53 *gene (17p13.1) and a control probe for the centromere of chromosome 17 (Vysis, Downers Grove, IL) were applied onto each sample, and fluorescent images corresponding to DAPI, SpectrumGreen (CEP17), and SpectrumOrange (17p13.1) were sequentially captured using the same equipment described for cCGH analysis. Only intact, non-overlapping nuclei were scored. An abnormal population was considered representative when at least three nuclei within the same microscope field presented a given aberration and at least 40 nuclei presented that particular alteration in the whole sample.

### TP53 mutation status

From the 51 samples subject to mutation analysis, direct sequencing (sense and anti-sense) was performed for each of exons 5–8 in 14 samples. The remaining 37 samples were screened for aberrant bands using the temporal temperature gradient electrophoresis (TTGE) method for exons 5, 6, and 8, whereas exon 7 was directly sequenced. The TTGE method has a better resolution level than sequencing, and aberrant bands may be detected in a sample with <5% mutated alleles [44].

## Competing interests

The author(s) declare that they have no competing interests.

## Authors' contributions

FRR carried out the microarray experiments, performed data analysis, and drafted the manuscript. RH collected and graded all prostate carcinomas, gathered clinical information for these patients, and assisted in drafting the manuscript. CJ assisted with sample DNA extraction and drafting the manuscript. MB assisted with the microarray experiments and data analysis. MH performed the mutation screening of the *TP53 *gene. MRT and RAL participated in the design and coordination of the study, assisted with analysis, and contributed to manuscript writing. All authors read and approved the final manuscript.

## Supplementary Material

Additional File 1 Comparison of cCGH and aCGH findings in 46 prostate cancer samples. Detailed description of cCGH and aCGH findings for each individual sample analysed in this study. Click here for file
