# Supplementary material for: Comparison of chromosomal and array-based comparative genomic hybridization for the detection of genomic imbalances in primary prostate carcinomas
Source: Mol Cancer. 2006 Sep 4;5:33. doi: 10.1186/1476-4598-5-33 (PMC1570364; doi:10.1186/1476-4598-5-33)
Supplement: Additional File 1 — Comparison of cCGH and aCGH findings in 46 prostate cancer samples. Detailed description of cCGH and aCGH findings for each individual sample analysed in this study. [file 1476-4598-5-33-S1.pdf]

Supplementary Table 1. Comparison of cCGH and aCGH findings in 46 prostate cancer samples

ID*		Chromosomal CGH	Array-CGH
Bp 1a	enh	8q	8p11.1-8q24.3(85),22q11.1-22q11.21(3),22q12.3-22q13.2(14)
	dim	2q11q12,8p,16q	2q11.2-2q12.1(5),5q13.2-5q13.3(5),6q14.1-6q14.3(5),8p23.3-8p11.21(53),16q12.1-16q14.3(53),17p13.2-17p13.1(9),22q11.21-22q12.3(17),22q13.2-22q13.33(11)
Bp 4	enh	8q	8q11.1-8q24.3(83)
	dim	2q21q31,6q13q16,8p,16q21qter,18q12q21	2q14.3-2q32.3(66),3q29(7),5q13.1-5q13.2(5),6q12-6q15(20),8p23.3-8p11.1(53),10p11.23-10p11.22(2),10q23.31(4),10q25.2-10q25.3(3),10q26.13-10q26.3(11),11p15.5-11p15.4(7),13q32.1-13q32.3(3),14q21.3-14q22.3(10),16q12.2-16q24.3(45),17p13.2-17p13.1(6),18q11.2-18q21.31(31)
Bp 12a	enh		
	dim		1q23.1-1q31.2(61),6q14.2-6q16.1(13),10q22.3-10q23.31(11),21q22.2-21q22.3(8)
Bp 13a	enh	5p15,7p,7q21qter,20p12pter,20q13q13	7p22.3-7p12.3(58),7p12.1-7q36.3(122),20p13-20q13.33(76)
	dim	5q13q31,6q14q22,8p21,13q14q31	3q12.1(2),5q11.2-5q31.1(87),6q12-6q22.32(60),7p12.2-7p12.1(2),8p23.3-8p12(45),12p13.2-12p12.3(7),13q13.1-13q31.1(40)
Bp 16	enh	3p23pter,3q25q28,8q21qter	3p26.3-3p21.31(62),3q11.2-3q29(132),5q13.2(3),8p11.21-8q24.23(78),15q11.2-15q13.2(7),15q22.2-15q26.3(48)
	dim	8p12p22,10q21q24,17p13	8p23.3-8p11.21(51),8q24.23-8q24.3(2),10q21.2-10q25.3(54),12q24.31-12q24.33(12), 17p13.3-17p11.2(25)
Bp 22	enh	7p15pter,7q21qter	7p22.3-7q36.3(181)
	dim	5q14q31,6q13q22,8p12p22,13q12q22	5q14.3-5q23.2(39),6q11.1-6q22.31(58),8p22-8p12(24),8q23.3-8q24.11(4),13q13.2-13q31.1(38),16q13-16q21(3),19p13.3-19q13.43(63),22q11.1-22q13.33(45)
Bp 26a	enh	5p13pter,7,8q,13q12,13q32q34,17p11p12	1p32.1-1q31.3(77),5p15.33-5p12(41),5q35.3(2),7p22.3-7q36.3(149),8q11.1-8q24.3(66),9p24.1-9q34.3(63),17p11.2(5)
	dim	5q11q13,8p,13q14q21,17p13	5q11.1-5q14.1(27),8p23.3-8p11.21(49),17p13.3-17p12(16)
Bp 28	enh	1q,6q23q26,7q11q22,8q13qter,11q13q23, 16p12,17q23q25	1q21.1-1q44(78),6q24.1-6q25.3(18),7q11.21-7q22.1(42),8q13.2-8q24.3(62),11q13.2-11q23.1(46),16p13.3-16p11.2(36),17q23.2-17q25.3(22),19p13.3-19p13.11(7)
	dim	8p12p23,13q21q31,16q	1p21.2-1p12(17),5q11.1-5q11.2(10),5q13.1(2),6q25.3(6),8p23.3-8p11.21(53),13q12.3-13q34(68),16q11.2-16q24.3(52),17q21.31(2),21q22.2-21q22.3(5)
Bp 36a	enh		
	dim		
Bp 38	enh		
	dim	5q11q13,5q21q23,8p,10q22q25, 12q22q23,17p	4q22.3(2),5q11.1-5q23.3(82),5q32-5q33.3(14),6p25.3-6p22.1(32),8p23.2-8p11.1(47),10q22.3-10q26.12(45),11q22.3-11q23.1(7),12p13.31-12q13.11(38),12q21.32-12q24.1(24),12q24.31-12q24.33(13),16q12.1-16q21(11),16q23.3-16q24.1(7),17p13.3-17p11.2(25),20q11.21-20q11.22(3)
Bp 41a	enh		3p26.3-3q29(229),7p22.3-7q36.3(178),9p24.2-9q34.3(93),20p13-20q13.33(72)
	dim		5q21.1-5q23.1(21),6q13-6q22.31(52),8p22-8p12(19),13q14.11-13q14.13(6)

Bp 49	enh	7q31,8p12qter,17q24q25	8p21.1-8q24.3(104),15q11.2-15q13.3(8),15q15.1-15q21.2(13)
	dim	5q11q21,8p22pter,10q11.2-10q21, 10q22q24,13q,16q,17p13	5q11.2-5q12.1(10),5q14.1-5q21.1(24),8p23.3-8p21.2(33),10p12.1(2),10q11.21-10q11.23(9),10q22.3-10q25.1(33),11p13(2),12p13.33-12q24.33(147),13q12.11-13q34(78),14q23.2-14q32.2(26),15q13.3-15q14(6),15q21.2-15q26.3(57),16q12.1-16q24.3(53),17p13.3-17p13.1(13),20p12.3-20p11.23(14)
Bp 52	enh		1q32.1(7),2q23.3-2q31.1(25),3p24.2-3p22.1(20),3q13.31-3q29(110),4q13.3(2),5q13.2(3),8p23.1(3),8q24.13-8q24.21(3)
	dim		6p25.1-6p24.3(9),6q14.1-6q22.31(35),8q24.3(3),13q21.32-13q21.33(4)
Bp 54a	enh	5p15,7q31,10q21,21q22	
	dim	1p21p31,5q11q13,6q16q22,8p12p22	1p31.1-1p21.2(33),2q22.1-2q23.3(12),3q13.31-3q21.1(13),4p15.1-4p12(15),5q11.1-5q14.1(40),5q14.3-5q21.3(17),6q14.1-6q22.33(46),6q25.3-6q26(10),8p22-8p12(13),8q24.11-8q24.3(25),11q12.1(2),13q31.3-13q33.1(8),16q21-16q22.1(7)
Bp 56	enh		19p13.3(3)
	dim		5q13.3-5q23.1(20),8p23.3-8p12(36)
Bp 63	enh	1q22q32,5p14pter,8p11p12,8q	1q12-1q44(82),6p22.3-6p21.1(22),6q24.1-6q27(28),7q36.3(3),8p12(4),8q11.1-8q24.3(84),9q22.2-9q22.31(2),11q25(3)
	dim	8p21pter,10p12pter,12p13,13q13q14, 14q11q12,16q23qter	1p36.33-1p13.1(123),2p25.3-2q37.1(248),3p12.2-3q21.3(47),5q11.1-5q34(117),6p25.3-6p22.3(21),6p12.1-6q24(77),8p23.3-8p12(44),8p11.21-8p11.1(6),9p24.2-9q22.1(43),9q22.31-9q34.3(46),10p15.3-10q26.3(124),11q25(2),12p13.33-12p11.21(35),13q12.3-13q21.1(24),14q11.2-14q32.33(92),15q13.3-15q26.3(76),16q22.1-16q24.3(24),17q11.2-17q25.3(72),18q11.2-18q23(58),19p13.3(4),21q22.2(3),22q11.1-22q13.33(45)
Bp 66	enh	3q13qter,4q21q22,7q31q36	3q13.31-3q29(108),4q13.3-4q22.1(16),7p22.3-7q36.3(176)
	dim	1p22p31,5q23,6q16q22,13q14,15q21	1p31.1-1p21.2(30),2p23.2-2p23.1(3),4q28.2-4q31.21(8),5q21.1(2),5q22.1-5q23.2(17),6q14.3-6q22.31(37),8p22-8p12(23),10q21.3(2),12p13.32-12p12.3(11),12q12(2),13q13.3-13q14.3(14),13q31.2(2),15q14-15q21.3(24),22q11.21-22q13.32(43)
Bp 71	enh		7q11.21-7q11.23(15),9q21.11-9q34.2(51)
	dim		6q14.1-6q16.1(14),9p24.2-9p11.2(25),10q26.2-10q26.3(4),13q12.11-13q34(65),17p13.3-17q21.33(59),18q23(3)
Bp 72	enh	8q21qter	8p11.21-8q24.3(89)
	dim	8p12pter,10q22q23,12p,16q23qter, 17p12pter,22q21q22	5q12.3-5q13.2(8),8p23.3-8p12(37),8p12-8p11.21(9),10q22.1-10q24.32(36),11q23.2-11q23.3(4),12p13.33-12p11.21(34),16q22.1-16q24.3(27),17p13.3-17q25.3(100),20q11.21-20q11.23(7),21q21.3-21q22.3(14),22q11.21-22q13.33(44)
Bp 73	enh	8q13qter	8q12.3-8q24.3(59),12q15-12q21.2(11),14q24.3-14q32.2(28)
	dim	13q14q31	1p36.33-1p32.2(66),2q11.1-2q23.3(57),2q37.1-2q37.3(9),4p16.3-4p15.2(21),7p22.3(3),8p23.3-8p23.2(6),13q13.1-13q31.1(31),14q32.2-14q32.33(8),16q22.2-16q24.3(23),17p13.3-17q25.3(96),18q21.1-18q23(35),19p13.3-19q13.43(61)
Bp 82a	enh	1p12p31,1q,3q21q26,7,8q,17q22qter	1p31.3-1q44(179),3p12.1-3q26.32(105),7p22.3-7q11.22(76),7q21.11-7q22.2(29),7q31.1-7q34(42),7q36.1-7q36.3(11),8p23.3-8p23.2(13),8p11.21-8q24.3(84),9q21.33-9q34.3(54),17q21.32-17q25.3(42),20p13-20q13.33(74)
	dim	3q28qter,6q15q23,8p12p22,9q21	1p32.3-1p31.3(7),2q37.1-2q37.3(10),3p24.3-3p24.2(7),3p13-3p12.2(12),3q26.32-3q29(27),6q14.1-6q22.33(48),7q22.3-7q31.1(5),7q35-7q36.1(9),8p22-8p11.21(33),9p24.2-9p23(12),10p15.3-10q26.3(128),12p13.2(2),16q12.1-16q12.2(5),16q22.2-16q22.3(5),17p13.3-17q21.32(58),21q11.2-21q22.3(32)
P 03	enh		
	dim	5q23q31,8p21p22	5q22.2-5q32(44),8p23.3-8p12(42),12p13.33-12p12.3(19)
P 04	enh	5p14pter,5q11q23,5q32q33	5p15.33-5q35.3(181)
	dim		3p21.32-3p21.31(7),8p21.3-8p21.2(8)

P 05	enh	8q	5q13.2(3),8p11.1-8q24.3(82)
	dim	2q23q24,8p12p23,10p11p12,10q22q25	2q11.2-2q32.1(86),8p23.3-8p11.21(52),10p15.3-10q26.3(121),16p12.2-16p12.1(5),17p13.3-17p11.2(23)
P 14	enh		
	dim		16q22.1-16q24.3(23),17p13.3-17p13.1(10)
P 15	enh		
	dim		
P 16	enh		
	dim		8p21.2-8p12(12)
P 20	enh		
	dim		
P 22	enh		1p31.3-1p31.1(14),5p15.33-5q13.3(78),7p22.3-7q31.31(129),8p11.21-8q11.1(3)
	dim	8p12pter	8p23.3-8p11.21(52)
P 23	enh		
	dim		
P 25	enh		
	dim		
P 28	enh		
	dim	5q21q23,8p21p22	2q14.3-2q21.3(12),5q14.3-5q23.3(43),8p22-8p21.2(13),12p13.33-12p11.21(34),12q24.31-12q24.33(12)
P 29	enh		
	dim		
P 33	enh		
	dim	8p12pter	3p14.1-3p12.3(8),5q12.3-5q14.1(10),5q23.2-5q31.1(15),8p23.3-8p11.21(43),12p13.33-12q14.1(40),17q21.31(2),22q12.1-22q12.3(12)
P 35	enh		
	dim	8p21pter	1p36.33-1p34.2(46),1q32.1-1q32.2(4),2q11.2-2q13(15),8p23.3-8p11.1(51),12q23.3-12q24.33(24),13q13.3-13q14.3(17),14q31.1-14q32.13(17),16q22.1-16q24.3(28),17q21.2-17q21.31(7),19q13.2-19q13.33(12),21q21.1-21q22.3(30)
P 36	enh		

	dim	16q22qter	16q22.1-16q24.3(21)
P 38	enh		
	dim	8p12p22	6q25.3-6q27(11),8p22-8p11.1(30),10q23.31(4),20p13-20p11.23(19)
P 41	enh	18p11	1p13.1-1q42.11(76),8p12-8q22.3(31),21q21.1-21q21.2(4)
	dim	8p22pter,13q14q22	8p23.3-8p12(24),13q12.13-13q34(14)
P 43	enh		
	dim	6q15q22	6q14.1-6q23.2(40)
P 45	enh		
	dim	8p12p22,17p12pter	8p23.3-8p11.21(47),17p13.3-17p11.2(21)
P 46	enh		
	dim		
P 48	enh		
	dim		
P 49	enh	3q23q26,7p13p21,7q21q32,8q21q24	3p26.3-3p26.2(4),3q13.13-3q29(117),7p22.3-7q36.3(179),8p12-8q11.1(13),8q12.2-8q24.3(69)
	dim	16q22qter	1p34.1-1p33(7),1q32.3-1q44(29),5q11.1-5q22.1(59),6q12-6q16.1(28),6q24.2-6q27(27), 8p22-8p12(23),16q11.2-16q24.1(49)
P 50	enh		
	dim	8p12p22,10q22qter,13q14q21,16q23q24	8p23.3-8q11.21(57),10q22.3-10q26.3(57),13q12.11-13q22.2(45),16q22.2-16q24.3(23)
P 51	enh	8q21q24	1q21.1-1q25.1(9),8q11.1-8q24.3(57),9p24-9q34.13(54)
	dim	8p22	8p23.3-8p11.21(45),12p13.2(2),16q22.2-16q24.3(19)

*Sample ID corresponds to the original cCGH publications (Ribeiro *et al.* 2006a for prostatectomy samples, and Ribeiro *et al.* 2006b for biopsy samples). The number of clones involved is provided for all aCGH findings, which were obtained with the software “Array-CGH Smooth”. Amplifications and homozygous deletions are not included, as these are provided in Table 2. Abbreviations: enh – enhanced; dim – diminished.
